# Benzodiazepine Use During Hospitalization: Automated Identification of Potential Medication Errors and Systematic Assessment of Preventable Adverse Events

**DOI:** 10.1371/journal.pone.0163224

**Published:** 2016-10-06

**Authors:** David Franklin Niedrig, Liesa Hoppe, Sarah Mächler, Heike Russmann, Stefan Russmann

**Affiliations:** 1 Department of Clinical Pharmacology and Toxicology, University Hospital Zurich, Zurich ZH, Switzerland; 2 Swiss Federal Institute of Technology Zurich (ETHZ), Zurich, ZH Switzerland; 3 drugsafety.ch, Küsnacht ZH, Switzerland; 4 Neurologie am See, Küsnacht ZH, Switzerland; University of Manitoba, CANADA

## Abstract

**Objective:**

Benzodiazepines and “Z-drug” GABA-receptor modulators (BDZ) are among the most frequently used drugs in hospitals. Adverse drug events (ADE) associated with BDZ can be the result of preventable medication errors (ME) related to dosing, drug interactions and comorbidities. The present study evaluated inpatient use of BDZ and related ME and ADE.

**Methods:**

We conducted an observational study within a pharmacoepidemiological database derived from the clinical information system of a tertiary care hospital. We developed algorithms that identified dosing errors and interacting comedication for all administered BDZ. Associated ADE and risk factors were validated in medical records.

**Results:**

Among 53,081 patients contributing 495,813 patient-days BDZ were administered to 25,626 patients (48.3%) on 115,150 patient-days (23.2%). We identified 3,372 patient-days (2.9%) with comedication that inhibits BDZ metabolism, and 1,197 (1.0%) with lorazepam administration in severe renal impairment. After validation we classified 134, 56, 12, and 3 cases involving lorazepam, zolpidem, midazolam and triazolam, respectively, as clinically relevant ME. Among those there were 23 cases with associated adverse drug events, including severe CNS-depression, falls with subsequent injuries and severe dyspnea. Causality for BDZ was formally assessed as ‘possible’ or ‘probable’ in 20 of those cases. Four cases with ME and associated severe ADE required administration of the BDZ antagonist flumazenil.

**Conclusions:**

BDZ use was remarkably high in the studied setting, frequently involved potential ME related to dosing, co-medication and comorbidities, and rarely cases with associated ADE. We propose the implementation of automated ME screening and validation for the prevention of BDZ-related ADE.

## Introduction

Benzodiazepines and “Z-drug” GABA-receptor modulators (BDZ) are among the most frequently used drugs worldwide [1–3]. Most BDZ have labeled indications for anxiety and sleeping disorders [3, 4]. BDZ are also used as add-on therapy for psychiatric disorders, pre-operative sedation, and the prevention and treatment of seizures. They are frequently prescribed in hospitals, institutions and community dwelling settings, and they feature a wide therapeutic range [5, 6]. According to their summary of product characteristics (SPC), BDZ are not intended for long-term use. However, long-term treatment with BDZ is frequent and may lead to tolerance and addiction [2, 3, 7]. Physical dependence and abuse are well known challenges which have resulted in health authorities and insurances often imposing special regulations with regard to BDZ prescribing, dispensing and compensation [8].

Severe adverse drug events (ADE) of BDZ, particularly at higher doses, include musculoskeletal weakness with falls and subsequent injuries [9–11], respiratory depression [12–15], paradoxical reactions [16–19] and CNS depression [4]. For differential diagnosis of a BDZ intoxication and the treatment of its symptoms, the antidote flumazenil can be administered to quickly antagonize the effects of BDZ [4]. Due to altered pharmacokinetics and increased intrinsic sensitivity, BDZ use can be particularly problematic in elderly and frail patients [20, 21]. Restrictive use of BDZ and low dosing upon treatment initiation is therefore recommended according to their labels and expert consensus guidelines such as the “Beers” and “Priscus” lists, or the “STOPP” criteria [4, 22–24]. Concomitantly administered drugs may reduce the metabolism of BDZ via inhibition of cytochrome P450 enzymes (CYP), leading to increased BDZ effects [25]. Strong CYP inhibitors may lead to a five- to tenfold increase in BDZ exposure, and some of these drug-drug-interactions (DDI) may result in dose-dependent adverse effects. Furthermore comorbidities such as acute renal impairment or respiratory disease can render patients more vulnerable to adverse effects of BDZ.

Prevalence of potential medication errors (ME) related to BDZ use has been studied before [7, 9, 26, 27]. For example, Zint et al. found that concomitant use of BDZ with certain CYP inhibitors was associated with an increased risk of hip fractures in a community dwelling setting [9]. However, there is a paucity of data on the clinical relevance and preventability of BDZ-related potential medication errors (ME) in tertiary care settings. Any failures in the drug treatment process that may cause harm to the patient are designated as medication errors (ME) [28]. They represent the most common preventable cause for ADE and are a major public health burden. While mistakes regarding storing and preparation of drugs are also considered ME, errors during the prescription or administration process account for about 90% of preventable ADE [29, 30]. Inadequate prescriptions, i.e. with risks clearly exceeding benefits, are of special interest: these decision-based ME are theoretically preventable by automated alerts triggered upon electronic prescription of the medication. In a tertiary care setting patients may frequently feature additional risk factors for BZD-induced ADEs related to polymorbidity and frailty, and may also be more often exposed to potent CYP inhibitors compared to patients in other settings.

In order to analyze and improve drug safety in a tertiary care hospital we had previously extracted electronic drug prescriptions, renal function measures and other clinical data from the database of an existing electronic clinical information system and set up a local pharmacoepidemiological database. This step is essential for two reasons. First, a rational allocation of limited available resources to improve hospital drug safety requires systematic retrospective real-life data on the frequency of preventable ME, and the usually much lower frequency of resulting severe ADE. In addition, local prescribers and decision makers may feel more compelled to act after being challenged with opportunities for improvement based on previous local ME. Second, currently available clinical decision support systems suffer from low specificity regarding clinical relevance of their alerts, leading to overalerting and consequent indiscriminate alert overriding. The analysis of local medication errors enables us to locally develop and implement customized highly specific alert algorithms. These require an interface with the local clinical information system as a necessary prerequisite for the development of real-time analyses and alerts that can be returned through the same interface to local safety experts and prescribers. Such locally optimized systems may eventually offer the necessary efficiency and efficacy in order to have a measurable impact on patient safety in clinical practice.

The current study aimed to evaluate the following outcomes: 1) BDZ usage patterns and frequency of potential ME, 2) frequency of ME due to inappropriate BDZ use as validated by the individual’s clinical records, 3) associated ADEs as a result of BDZ related MEs.

## Methods

### Study population, data collection and study design

**Fig 1:**
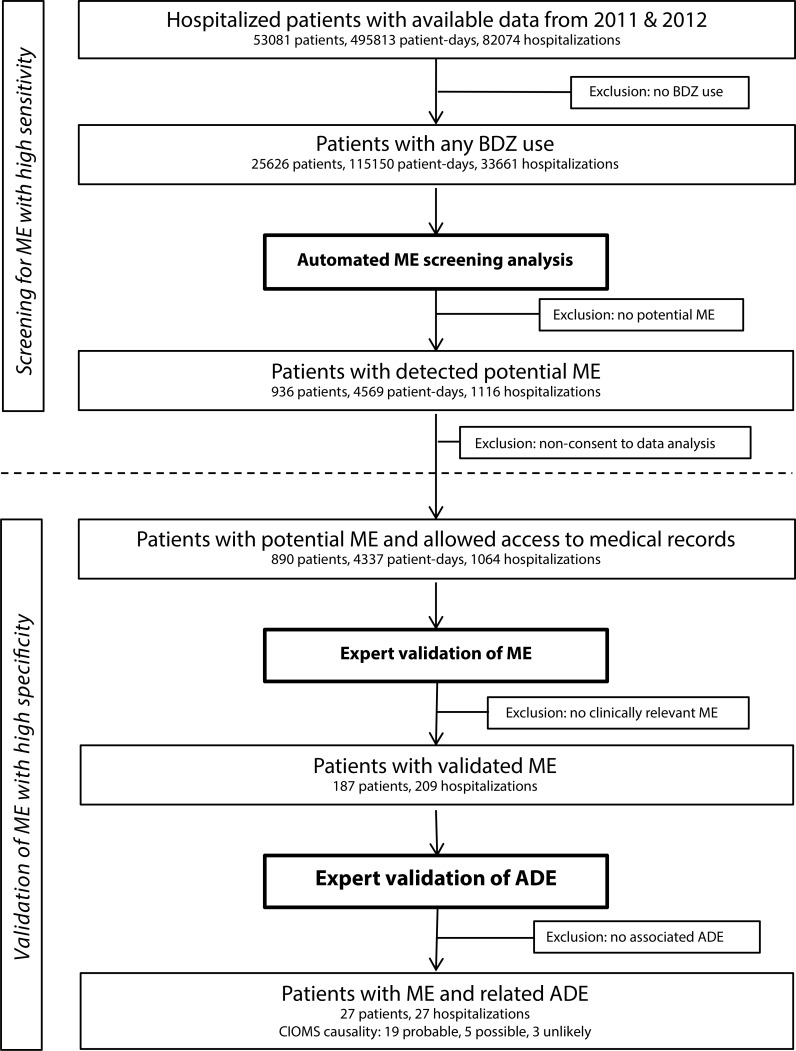
Study population and overall study design.

Selection of the study population and overall study design are presented in Fig 1. We conducted a retrospective observational study that analyzed BDZ usage patterns, potential ME, and associated ADE in a tertiary care teaching hospital with about 1,000 beds and 40 clinical specialty divisions providing health care to a catchment area of about 1.5 million individuals. The cantonal ethics committee of Zurich (Kantonale Ethikkommission Zürich, KEK-ZH-Nr. 2015–0268), the hospital’s medical director and the hospital’s center for clinical research had approved the data extraction, the setup and analysis of the anonymized and de-identified pharmacoepidemiological database, and the access to original medical records for our research. Patients that had disapproved the use of their data for research upon admission were excluded from the validation procedure with review of original medical records.

### Data Source

We used our previously described comprehensive pharmacoepidemiological database containing information on demographics, laboratory results and electronic drug prescriptions for hospitalized patients of a Swiss tertiary care hospital covering admissions from the calendar years 2011 and 2012 [31]. Our database builds on information extracted from the hospital’s electronic clinical information system featuring electronic drug prescription (computerized physician order entry). The system records not only prescriptions but also a confirmation for each drug’s actual administration to the patient along with its time. Our analyses included all prescriptions with documented administration from all hospitalized patients during the study period, except patients staying at intensive care units, where computerized physician order entry had not yet been introduced. ICD-10 codes of primary diagnoses were available for the calendar year 2012. For the validation of potential ME and ADE we had access to and reviewed comprehensive electronic medical records.

### Prevalence of BDZ use and algorithm-based identification of potential medication errors

We developed and programmed algorithms for the automated detection of patient-days with potential medication errors. Algorithms were customized and validated separately for each studied BDZ. Our analyses focused on frequently used BDZ with potentially relevant interactions via CYP metabolism or with altered pharmacokinetics in renal impairment and included the following drugs: zolpidem, midazolam, diazepam, alprazolam, triazolam, zopiclone, flunitrazepam, clorazepate, nitrazepam, prazepam and lorazepam (S1 Table: BDZ used in the study population and their potential for clinically relevant effects from CYP metabolism / renal impairment). Clobazam was not analyzed using automated algorithms because in the studied setting it is predominantly used for treating post-stroke epilepsy and delirium, two conditions where doses are individually titrated [4]. In order to identify BDZ administrations and potentially interacting co-medication we used Anatomical Therapeutic Chemical (ATC) codes provided by the World Health Organization (WHO) [32]. For each BDZ undergoing CYP-metabolism we established a list of relevant CYP inhibitors. These were based on the drugs’ summary of product characteristics (SPC) and comprehensive scientific information sources including specialized drug interaction databases and websites [33–36]. The final lists only included CYP-inhibiting drugs with a strong effect according to at least one reference. Certain strong CYP 3A4 inhibitors (e.g. clarithromycin) are known to irreversibly bind and inactivate CYP 3A4 enzymes, which results in reduced metabolic capacity until they are synthesized *de novo* [36, 37]. Other strong CYP 3A4 inhibitors do not exhibit such a mechanism-based effect (e.g. itraconazole), but their metabolites may continue to inhibit CYP metabolism for some time after cessation [38, 39]. Hence administration of strong CYP 3A4 inhibitors was considered potentially relevant if it occurred on the same day or up to two days prior to BDZ administration and thus defined our search algorithms for patient-days on which BDZ administration represented a potential ME. Furthermore, we also identified additional potential risk factors for BDZ associated ADE, i.e. concomitant use of multiple BDZ, additional relevant CYP inhibitors of CYP 2C19 or 1A2, and co-medication of opioids and muscle relaxants on the same day [40–42]. A list of the considered CYP inhibitors is presented in the S2 Table: CYP inhibitors considered for potentially relevant drug-drug interactions. The presence of these factors per se was not considered a ME but they were assessed in order to validate the detected ME.

Because severe renal impairment is a formal contraindication to the use of lorazepam according to the Swiss SPC, we developed an algorithm that identified all patients with lorazepam administrations and a current eGFR < 30 ml/min according to the CKD-EPI formula. A current eGFR was defined as a creatinine measurement within 72 hours before the administration of lorazepam Furthermore, we also identified and validated any use of the specific BDZ antidote flumazenil as a possible indicator of BDZ-related ME.

### Validation of medication errors (ME)

For every hospitalization, during which at least one day with a potential ME was identified using the algorithms described above, we validated the clinical context of the BDZ administrations. For that purpose we reviewed the original medical records and compiled the following additional information: indication, dose and route of administration of any BDZ, concomitant use of opioids and muscle relaxants, long-term oxygen therapy before and after BDZ administration, alcohol and substance abuse, relevant severe pulmonary and liver diseases, organ transplantation, renal replacement therapy, and whether the BDZ was administered in a palliative situation. Finally, we assessed the clinical relevance of these parameters for each patient’s individual clinical situation and determined whether BDZ administration was a validated ME, i.e. a more cautious or no use of BDZ with alternative therapy would have been indicated under the given circumstances (Table 1**).**

**Table 1:** Qualitative impact of patient parameters on validation of ME.

*Parameter*	*Impact on assessment of ME*	
Palliative situation	Benefit outweighs risk, no ME	✖
Known BDZ abuse	Tolerance of high BDZ dose, no ME	✖
Low dose of BDZ	≤ 1/2 of standard dose	**↓**
≥ 1 other BDZ	depending on number & dose	**↗**
Respiratory insufficiency	If severe	**↗**
Hepatic impairment	If severe	**↗**
Age ≥ 65 & dose	If initial dose not reduced	**↗**

Examples: A hospitalizations featuring a patient that receives the standard-dose of a BDZ metabolized by CYP 3A4 with a co-administered of a strong CYP 3A4 inhibitor and two other BDZ while suffering from respiratory insufficiency qualifies as a validated ME unless the BDZ were administered in a palliative situation. However, if a patient was exposed to one BDZ and a corresponding CYP interaction but the dose of the BDZ was ≤ 1/2 of the standard dose the hospitalization is not contributing to validated ME.

✖ Hospitalization with patient parameters that suggest no medication errors (ME) occurred

**↗** Hospitalization with patient parameters that require a careful benefit / risk assessment of any BDZ use

**↓** Hospitalization features careful dosing, therefore ME unlikely

### Identification and assessment of adverse drug events (ADE)

For all validated ME we reviewed comprehensive original medical records for documented associated ADE including falls, severe and prolonged CNS depression, respiratory depression, apnea, paradoxical reactions, hypoxemia and coma. If an associated ADE was documented we assessed the causal relationship using standardized international WHO / Council for International Organizations of Medical Sciences (CIOMS) causality assessment criteria which is the established worldwide standard method used by regulatory agencies and industry [43]. We also assessed whether the associated ADE may have been preventable, i.e. if the following of our recommendations would have been communicated and had been followed by the prescribers: lower initial BDZ dose, omission of additional BDZ, reducing the amount of “on demand” BDZ prescriptions, using a different BDZ without relevant CYP metabolism or one that can be used in renal impairment.

### Data analysis

Data analysis was descriptive with presentation of results in tables as appropriate. Frequencies were calculated with regard to individual patients, hospitalizations and patient-days. Data management and analyses were done using STATA Version 13.1 (STATA Corporation, College Station, TX, USA).

## Results

### Prevalence of BDZ use

Among a source population of 53,081 individual patients contributing 82,074 hospitalizations and 495,813 patient-days, we identified the study population of all BDZ users. Frequency of BDZ use, demographics and other characteristics of the study population are presented in Table 2. BDZ were administered on 23.2% of all patient-days, and at least once in 48.3% of all hospitalized patients and in 41.0% of all hospitalizations. Mean duration of hospitalization was 10 days (median: 5 days, range 0–397 days). On 42.0% of patient-days with any BDZ use 11 or more additional drugs were administered during hospitalization.

**Table 2:** Characteristics of the study population.

	*patient-days*		*patients*		*hospitalizations*	
	*n*	*%*	*n*	*%*	*n*	*%*
All patients hospitalized in 2011 & 2012	495,813		53,081		82,074	
**Study population**						
Administration of ≥ 1 BDZ	115,150	100	25,626	100	33,661	100
**Age**						
< 18	1,037	0.9	677	2.6	803	2.4
18–44	23,831	20.7	7,903	30.8	9,701	28.8
45–64	42,938	37.3	8,565	33.4	11,632	34.6
65–85	42,736	37.1	7,608	29.7	10,450	31.0
> 85	4,608	4.0	873	3.4	1,075	3.2
**Sex**						
Male	58,566	50.9	12,860	50.2	17,225	51.2
Female	56,584	49.1	12,766	49.8	16,436	48.8
**# of concomitant drugs** ^**I**^						
1–5	24,113	20.9	8,419	32.9	11,430	34.0
6–10	42,723	37.1	7,530	29.4	10,138	30.1
11–20	44,861	39.0	6,360	24.8	9,007	26.8
≥ 21	3,453	3.0	3,317	12.9	3,086	9.2
**Most frequently administered BDZ** ^**II**^						
Lorazepam	41,540	36.1	8,704	34.0	10,753	31.9
Zolpidem	34,841	30.3	7,150	27.9	8,873	26.4
Midazolam	20,362	17.7	14,993	58.5	17,549	52.1
Oxazepam	11,370	9.9	2,487	9.7	2,929	8.7
Clobazam	5,285	4.6	617	2.4	760	2.3
Bromazepam	3,232	2.8	453	1.8	541	1.6
Diazepam	1,736	1.5	324	1.3	402	1.2
Alprazolam	1,586	1.4	184	0.7	238	0.7
Clonazepam	1,549	1.3	196	0.8	248	0.7
Triazolam	569	0.5	48	0.2	75	0.2
**Administration of ≥ 2 different BDZ**						
Use of 2 BDZ	8,119	7.1	2,887	11.3	3,339	9.9
Use of 3 BDZ	386	0.3	205	0.8	221	5.9
Use of ≥ 4 BDZ	23	< 0.1	17	0.1	17	< 0.1
**Concomitant use of opioids**						
Use of 1 opioid	26,351	22.9	6,505	25.4	7,976	23.7
Use of 2 opioids	4,953	4.3	1,725	6.7	1,978	5.9
Use of 3 opioids	16	< 0.1	11	< 0.1	12	< 0.1
**Concomitant use of muscle relaxants**	1,860	1.6	322	1.3	1.3	1.1

^I^ Number of concomitant drugs was defined by individual ATC codes.

^II^ Considers use of multiple BDZ (benzodiazepines) on same patient-day.

Most frequently coded primary diagnoses for hospitalizations in 2012 with exposure to BDZ were “other forms of heart disease” (I30-I52, 4.1%), “benign neoplasms, except benign neuroendocrine tumors” (D10-D36, 4.1%) and “non-inflammatory disorders of female genital tract” (N80-N98, 3.1%). While lorazepam was the most frequently administered drug regarding patient-days (36.1%), midazolam was the most frequently used BDZ regarding individual patients (58.5%) and hospitalizations (52.1%). Zolpidem and oxazepam were also among the most frequently used BDZ. The use of some BDZ was marginally low, i.e. flurazepam, zopiclone, flunitrazepam, clorazepate, lormetazepam, nitrazepam, temazepam, prazepam and ketazolam accounted for only 1.4% of all BDZ administrations. For zolpidem, pharmacokinetics and pharmacodynamics change with age, and the recommended standard-dose is therefore reduced to 5 mg instead of 10 mg in patients ≥65 years of age. It should only be exceeded if efficacy is insufficient with 5 mg according to the SPC [4]. Therefore, the age- and dose-distributions for zolpidem users are of particular interest. A marginally higher proportion of patient-days with zolpidem use occurred in patients ≥65 years of age compared to all BDZ users (45.8% vs. 41.1%, respectively). Among all patient-days with zolpidem use the daily dose was at least 10 mg per day in 74.2%. For the subpopulation of patients ≥65 years this proportion was almost as high, i.e. 67.4%. This corresponds to an absolute number of 10,749 days of zolpidem use with a dose ≥10 mg per day in patients ≥65 years in the studied population or approximately 5,000 patient-days each year.

Administration of two different BDZ on the same day occurred in 7.1% of patient-days, and 211 patients received three or more different BDZ on the same day, with a maximum of 5 different BDZ. Co-medication with opioids was common (27.2% of patient-days on any BDZ), and on 4.3% of the studied patient-days two or more opioids were administered concomitantly. The departments with the highest proportion of BDZ use were the departments of reproductive endocrinology (45.4% of all patient-days), diagnostic and interventional radiology (34.7% of all patient-days), and radio-oncology (33.7% of all patient-days).

### Drug interactions, renal impairment and potential medication errors

We identified 19 different BDZ that were administered in the studied setting of a tertiary care hospital. For 9 of those we identified possible clinically relevant CYP-related DDI, and for lorazepam we identified critical use in severe renal impairment. Hospitalizations featuring exposure to these BDZ or the administration of lorazepam in patients with severe renal impairment were then further investigated for validation of the potential ME and are presented in Table 3. Overall, our algorithms detected such potential ME on 4,237 patient-days, occurring during 1,066 hospitalizations. This number is equivalent to an average of 5.8 potential ME regarding BDZ administrations on each calendar day in 2011 and 2012. Thereof 3,372 patient-days with potential ME were due to concomitant administration of BDZ with strong CYP inhibitors. With the exception of midazolam, BDZ that were used more frequently also contributed more hospitalizations with potential ME. Most were contributed by zolpidem (2,555 patient-days, 7.3% of all zolpidem patient-days) and midazolam (440 patient days, 2.2% of all midazolam patient-days). Alprazolam contributed only 191 patient-days but was the BDZ with the highest proportion of potential ME (12.0%). In addition we identified 1,197 patient-days with potentially inadequate administrations of lorazepam in patients with severe renal impairment representing 28.3% of all identified potential ME. Analyses of flumazenil use identified 15 patient-days (occurring during 13 hospitalizations) with potential ME related to BDZ use.

**Table 3:** Algorithm-based identification of potential ME.

	*Mechanism for potential ME*	*BDZ total use*		*Potential ME*			
		*patient-days*	*hospitalizations*	*patient-days*	*%*	*hospitalizations*	*%*
Zolpidem	Co-medication with ≥ 1 strong CYP 3A4 inhibitor	34,841	8,873	2,555	7.3	493	5.6
Midazolam^I^		1,401 /18,989	17,549	108 / 332	7.7 / 1.7	192	2.5
Diazepam		1,736	402	106	6.1	15	3.7
Alprazolam		1,586	238	191	12.0	16	6.7
Triazolam		569	75	9	1.6	3	4.0
Zopiclone		498	93	9	1.8	2	2.2
Flunitrazepam		349	75	38	10.9	7	9.3
Clorazepate		202	31	9	4.5	1	3.2
Nitrazepam		145	20	0	-	0	-
Prazepam^II^		48	9	0	-	0	-
Lorazepam	Severe renal impairment^III^	41,540	10,753	1,197	2.9	324	3.0
Flumazenil	BDZ antidote (surrogate for BDZ overdose)	15	13	15	100.0	13	100.0

^I^ Patient-days with p.o. / i.v. administration; hospitalizations with any i.v. or p.o. midazolam administration

^II^ For prazepam the co-administration of a strong or moderate CYP 2C19 inhibitor would also have been deemed a potential ME, however we did not detect any such combination

^III^ In order to qualify as potential ME, in addition to eGFR < 30 ml/min, patients hat to EITHER receive lorazepam on ≥ 2 consecutive days OR to also have had co-administered ≥ 1 additional BDZ

Our algorithms detected sporadic patient-days with exposure to numerous studied drugs, e.g. patients concomitantly receiving 2 BDZ *and* 2 strong CYP 3A4 inhibitors *and* 2 opioids *and* a muscle relaxant. Other patients received up to 510 mg zolpidem per day, but according to the medical records very high doses were prescribed intentionally in patients with severe BDZ addiction, and we therefore did not classify those as ME.

### Validation of medication errors

For 1,064 available hospitalizations we assessed the clinical relevance of algorithm-identified potential ME. After consideration of the patients’ individual clinical situation we classified 205 of those (19.3%) as validated ME (Table 4) which represents 0.25% of all hospitalizations in 2011 and 2012. Hospitalizations with potential ME concerning lorazepam use in severe renal impairment were classified as validated ME in 41.4%. Among potential ME in hospitalizations with exposure to zolpidem and strong CYP inhibitors, 56 (11.4%) were classified as validated ME. Most cases were assessed as clinically relevant due to pre-existing respiratory insufficiency and old age. Among hospitalizations with potential ME concerning midazolam, which is frequently administered only once before interventions, only 6.3% were confirmed as validated ME. For three hospitalizations with a potential ME concerning triazolam, all were confirmed as ME while none out of 15 potential ME with diazepam appeared clinically relevant, mostly because the patients were known drug addicts. Similarly, the assessment of the clinical context of potential ME with zopiclone, flunitrazepam and clorazepate did not contribute any validated ME.

**Table 4:** Potential and validated ME and associated ADE.

	*hospitalisations*		*≥ 1 other BDZ (n)*	*≥ 1 opioid (n)*	*Indication* ^*I*^ *(n)*	*Presence of risk factors (n)*		
	*n*	*%*			*sleep / anx / inv / unkn*	*respiratory insufficiency*	*severe liver disease*	*Age ≥65*
**Zolpidem potential ME**	**493**	**100**						
no ME	437	88.6	54	93	432 / 2 / 2 / 1	169	87	109
validated ME	56	11.4	6	10	55 / 0 / 0 / 1	27	6	38
validated ME & associated ADE	11	2.2	1	2	11 / 0 / 0 / 0	5	0	5
**Midazolam potential ME**	**192**	**100**						
no ME	180	93.8	47	51	6 / 24 / 145 / 5	52	18	34
validated ME	12	6.3	6	6	11 / 0 / 0 / 0	3	1	7
validated ME & associated ADE	1	0.5	0	1	1 / 0 / 0 / 0	0	0	1
**Triazolam potential ME**	**3**	**100**						
no ME	0	-	-	-	-	-	-	-
validated ME	3	100.0	1	1	3 / 0 / 0 / 0	1	0	2
validated ME & associated ADE	1	33.3	0	0	1 / 0 / 0 / 0	1	0	0
**Lorazepam potential ME**	**324**	**100**						
no ME	190	58.6	65	77	99 / 77 / 1 / 13	48	44	106
validated ME	134	41.4	25	39	100 / 31 / 1 / 2	49	27	82
validated ME & associated ADE	10	3.1	4	6	7 / 3 / 0 / 0	3	1	8

^I^ Indication: anx = anxiety / inv = pre-invasive / unkn = unkown

### Identification and assessment of adverse drug events

In the 205 hospitalizations where a validated ME had occurred we systematically searched the original medical records for related ADE. This revealed 23 patients with ADE compatible with intrinsic effects of BDZ, i.e. falls, prolonged CNS depression and dyspnea (Table 5). According to WHO/CIOMS causality assessment 15 of the ADE had a “probable”, 5 a “possible” and 3 an “unlikely” causal relation to the respective BDZ ME. Of the 10 ADE associated with lorazepam, 8 occurred in elderly patients and 7 were found to be preventable with a more cautious use—such as no co-administration of additional BDZ. Of the 11 ADE associated with zolpidem, 6 occurred in patients ≥65 years and 9 were found to be preventable. According to CIOMS criteria, one ADE associated with midazolam was assessed as ‘probable’ and preventable—it occurred in a patient with repeated oral administration of the drug despite presence of a strong CYP inhibitor and concomitant administration of another BDZ. On the other hand, causality of one ADE with tetrazepam was assessed as ‘possible’ and found not to be preventable during hospitalization, as BDZ administration and the ADE had actually occurred before admission. Finally, the identification of all flumazenil administrations in our dataset revealed four cases of severe ADE resulting from inadequate BDZ administrations during hospitalization. In two of those preventable ME strong CYP inhibitors had been co-administered with BDZ while in one case lorazepam had been administered in a patient with severe renal impairment (eGFR of 18 ml/min). Additional relevant CYP inhibitors and administration of multiple BDZ were also present in all four cases.

**Table 5:** Cases with severe ADE.

	*total numberADE*	*Fall*		*CNS depression*		*eGFR*^*I*^ *<30 ml/min*	*CIOMS*			*Presence of risk factors*			*Preventable*^*III*^	
		*minor injury*	*major injury*^*II*^		*→ with respir*. *depression*		*unlikely*	*possible*	*probable*	*respiratory insufficiency*	*severe liver disease*	*age ≥65*	*yes*	*no*
Zolpidem	11	5	2	4	2	1	2	1	8	5	-	5	9	2
Midazolam	1	1	-	-	-	-	-	-	1	-	-	1	1	-
Triazolam	1	-	1	-	-	-	-	1	-	1	-	-	-	1
Lorazepam	10	4	4	2	2	10	1	3	6	3	1	8	7	3
Flumazenil	4	-	1	3	1	1	-	-	4	2	-	2	4	-

^I^ Patients with preexisting impaired renal function. eGFR = estimated glomerular filtration rate according to CKD-EPI

^II^ Thereof four with subsequent emergency CT scans and one other case with fracture of femur

^III^ Indicates that automated algorithm would have been able to detect the ME and subsequent expert recommendation could have prevented it

## Conclusions

This study analyzed BDZ usage patterns, potential ME and associated ADE in the real-life setting of a tertiary care university hospital The use of BDZ as well as the risk for drug interactions was remarkably high: 48% of all hospitalized patients received a BDZ during hospitalization and the prevalence of BDZ use for all patient-days was 23%; concomitant exposure to two or more BDZ is rarely justified but was detected in 7% of patients; and ‘hyperpolypharmacy’ as defined by Onder et al. with more than 10 additional concomitant drugs was present in 42% of patient-days [44]. Some previous studies reported also high prevalences ranging from 10% to 30% [45–47], but these studies were much smaller and data on inpatient prescription patterns of BZD remains scarce. Overuse of psychotropic medication including BZD has also been criticized in other studies.

At the same time one has to realize that midazolam contributed 17.7% of patient-days with BDZ use, but midazolam was frequently only administered as a single intravenous dose before smaller diagnostic or therapeutic procedures. Of note, midazolam drug interactions with CYP inhibitors are much less important for intravenous as compared to oral administration with pronounced first-pass metabolism [4, 25].

For each studied BDZ, we applied an individually programmed algorithm that detected patient-days with potential ME. In addition to the co-administration of strong CYP 3A4 inhibitors, some patients were exposed concomitantly to inhibitors of CYP 2C19 or CYP 1A2. This may further reduce the capability to metabolize certain BDZ, (i.e. diazepam, clorazepate and prazepam for 2C19; zolpidem for 1A2) through the inhibition of a relevant metabolic bypass [3, 35]. Furthermore, the concomitant use of multiple opioids may further enhance CNS-related ADE of BDZ [40]. Algorithm-based detection of potential ME was highly efficient, but only clinically relevant prescribing errors should be considered as true ME and this distinction requires additional manual expert evaluation of individual patients using weighted information from non-structured data. Adapted dosing (i.e. less than half of the recommended dose), palliative situations, or known tolerance of high BDZ doses were the most frequent reasons why potential ME were considered as clinically irrelevant. At the other end of the spectrum current contraindicated conditions (e.g. severe respiratory failure), co-administration of multiple additional BDZ and lack of adaptation of the initial dose in elderly patients were the most frequent reasons why we assessed a potential ME as clinically relevant. Concerning lorazepam, the Swiss SPC lists a formal contraindication for use in severe renal impairment, whereas SPC in other countries contain less strict warnings. However, kinetic studies indicate that in case of repeated administrations in renal failure plasma concentration and half-life are not only prolonged for the inactive metabolite lorazepam glucuronide, but also for active lorazepam itself [48]. Of further note, 8 among 10 patients with severe renal impairment receiving lorazepam were also ≥65 years of age, for which the SPC recommends a 50% dose reduction regardless of renal function. Although there is no gold standard for expert validations of ME, we consider it as the best available method, and we have successfully applied and evaluated it in prospective studies and ward rounds with instant feedback from the prescribers and subsequent medication changes [49, 50].

Even a validated ME does not always result in a severe ADE, and although the proportion of ME that actually result in severe ADE is most important from a clinical point of view, this quantitative aspect is vastly understudied in drug safety research. Therefore, our study systematically searched for and quantified ADE following all validated ME. As expected, only a small proportion of ME led to severe ADE, but over a 2-year period we were able to identify the total number of 20 severe ADE following erroneous administrations of zolpidem, midazolam, triazolam or lorazepam with a formal causality assessment suggesting a causal role for these BDZ. Prospective screening for the underlying ME with our automated algorithms would have detected those, and timely alerts could therefore have effectively prevented them with high efficiency.

Furthermore, we also identified and assessed 15 patient-days with flumazenil administration, which revealed 4 cases of BDZ related ME that caused severe ADE requiring such antidote treatment. Three of those ME that had occurred during hospitalization would indeed have been detected in time by our algorithms, which identified co-administration of strong CYP inhibitors in two cases, and relevant renal impairment with an eGFR < 30 ml/min and concomitant exposure to an additional BDZ in another case. Only the remaining fourth case with flumazenil administration would not have been detected by our algorithms due to the lack of exposure to any strong CYP inhibitors. It involved the BDZ oxazepam and alprazolam concomitantly administered with the CYP 3A4 inhibitor fluconazole, which is considered to be only a moderate CYP 3A4 inhibitor [33, 35]. All four flumazenil cases could likely have been prevented by either choosing a lower dose of the involved BDZ or by using BDZ which are not affected by CYP inhibition. Of note, a study in a Brazilian teaching hospital analyzed the use of flumazenil in patients exposed to intravenous midazolam and interacting drugs [51]. They identified 23 patients exposed to clinically significant drug-drug interactions requiring administration of flumazenil during one year. Most of the cases were related to CNS depressing drugs such as opiates, whereas none were related to CYP 3A4 inhibitors, for which an interaction is much more pronounced if midazolam is administered orally.

Although our study focused on identifiable ME with BDZ one should also realize the high absolute use of BDZ in the studied setting, and that ADE to BDZ may also occur without preceding ME. Further interventions should therefore also promote a generally more restrictive use of BDZ use particularly in elderly patients and are supported by expert consensus guidelines such as the “Beers” and “Priscus” lists, or the “STOPP” criteria [4, 22–24].

Furthermore, our results showed that compliance with dose-adaptation recommendations for zolpidem in elderly patients is very low, i.e. two thirds of zolpidem users receive ≥10 mg/day. In combination with the high prevalence of zolpidem use this resulted in the high absolute number of 10,749 patient days with ≥10 mg of zolpidem use per day in patients ≥65 years over two years in a tertiary care hospital. An analysis of ADE resulting from all high zolpidem doses in elderly patients was beyond the scope of the current study, but our personal experience from safety ward rounds shows that most prescribers are not aware of recommended dose adaptations for zolpidem in elderly patients and readily change the dose when this is brought to their attention.

In conclusion BDZ use and related ME were remarkably high in the studied setting of a tertiary care hospital. Our algorithms are able to identify potential ME for BDZ prescriptions through an automated analysis of interacting co-medication and impaired renal function with high efficiency. Of note, concomitant use of barbiturates was not part of the screening algorithms used in the current study, and further ME related to their use with BDZ may have occurred.

Whereas the current study was performed retrospectively, our next aim is the implementation of prospective real-time screening algorithms for ME that issue immediate alerts. We found that about 20% of potential ME for BDZ prescriptions identified through our automated search algorithms were assessed as clinically relevant, i.e. BDZ prescriptions should have been changed. This further selection of clinically relevant ME still requires manual expert evaluation with a review of patients’ clinical situation and individual risk-benefit evaluation. However, if an automated algorithm performs the screening it would be an easy task for a trained expert to review the approximately 6 alerts that would be generated per day and subsequently recommend prescription changes in 1 to 2 patients per day. Furthermore, it is an integral part of our overall drug safety concept that any of our ME detection algorithms can be easily activated, deactivated or modified according to national and local requirements and preferences. Although serious ADE following ME with BDZ are fortunately rare, our findings indicate that such a system may prevent approximately 10 severe ADE per year in a tertiary care hospital. This absolute number is clinically relevant and may stand in a favorable relation to the resources that are required for the maintenance of a semi-automated proactive safety surveillance system. In addition, automated alerts for dose reduction of zolpidem in elderly patients and a generally more restrictive use of BZD may also prevent a considerable number of BDZ-related ADE and should be further investigated in future studies.

## Supporting Information

S1 Table BDZ used in the study population and their potential for clinically relevant effects from CYP metabolism / renal impairment. (DOCX)

S2 Table CYP inhibitors considered for potentially relevant drug-drug interactions. (DOCX)
