# Supplementary material for: Benzodiazepine Use During Hospitalization: Automated Identification of Potential Medication Errors and Systematic Assessment of Preventable Adverse Events
Source: PLoS One. 2016 Oct 6;11(10):e0163224. doi: 10.1371/journal.pone.0163224 (PMC5053537; doi:10.1371/journal.pone.0163224)
Supplement: S1 Table — (DOCX) [file pone.0163224.s001.docx]

S1 Table: BDZ used in the study population and their potential for clinically relevant effects from CYP metabolism / renal impairment

| **Drug** | **CYP-metabolism^1^** | **Half-life^2^** | **Special contraindications^3^** |
| --- | --- | --- | --- |
| flurazepam | x | 2,3 (40-100) h | chronic psychosis, phobic and obsessive states |
| prazepam | **3A4, 2C19** | 50-80 h |  |
| midazolam | **3A4** | 2 h | keto-/ itra-/ voriconazol, HIV-protease inhibitors (ritonavir) |
| triazolam | **3A** | 1,5-5,5 (4) h | azol-antimycotics, HIV-PI, psychiatric disorder |
| zopiclone | **3A4,** 2C8 | 6 h | chronic psychosis |
| bromazepam | 3A4, 2C9 | 20 h |  |
| nitrazepam | 3A4, 2D6 | 30 h | renal impairment, chronic psychosis, spinal und cerebellar ataxia, phobic and obsessive states |
| lormetazepam | x | 11-16 h |  |
| temazepam | 3A4 | 7-11 h | spinal und cerebellar ataxia |
| clonazepam | 3A4 | 30-40 h |  |
| flunitrazepam | **3A**, 2C19, 3A5 | 16-35 h | severe chronic hypercapnia, psychosis |
| oxazepam | x | 8 h |  |
| ketazolam | x | 2 (52) h |  |
| zolpidem | 3A4 | 3 h |  |
| lorazepam | x | 12-16 h | severe renal impairment |
| potassium clorazepate | x | 2,3 (30-150) h |  |
| clobazam | x | 36 (79) h |  |
| diazepam | x | 20-45 (96) h |  |
| alprazolam | x | 12-15 h | Azol-antimycotics, HIV-PI |

^1^ bold: main metabolic pathway / non-bold: secondary but relevant metabolic pathway

^2^ in brackets: half-life of active metabolites

^3^ all BDZ feature the following contraindications: BDZ-intolerance, myasthenia gravis, severe respiratory insufficiency, severe liver insufficiency (except oxacepam), sleep apnea
