# Supplementary material for: Benzodiazepine Use During Hospitalization: Automated Identification of Potential Medication Errors and Systematic Assessment of Preventable Adverse Events
Source: PLoS One. 2016 Oct 6;11(10):e0163224. doi: 10.1371/journal.pone.0163224 (PMC5053537; doi:10.1371/journal.pone.0163224)
Supplement: S2 Table — (DOCX) [file pone.0163224.s002.docx]

S2 Table: CYP inhibitors considered for potentially relevant drug-drug interactions

| drug | CYP |
| --- | --- |
| fluvoxamine | 1A2 |
| ciprofloxacin | 1A2 |
| enoxacin | 1A2 |
| ticlopidine | 1A2 |
| methoxsalen | 1A2 |
| pefloxacin | 1A2 |
| norfloxacin | 1A2 |
| grepafloxacin | 1A2 |
| fluvoxamine | 2C19 |
| esomeprazole | 2C20 |
| omeprazole | 2C21 |
| chloramphenicol | 2C22 |
| felbamate | 2C23 |
| fluoxetine | 2C24 |
| isoniazid | 2C25 |
| modafinil | 2C26 |
| oxcarbazepine | 2C27 |
| ticlopidine | 2C28 |
| topiramate | 2C29 |
| oral contraceptives | 2C30 |
| itraconazole | 3A4 |
| ketoconazole | 3A4 |
| nefazodone | 3A4 |
| saquinavir | 3A4 |
| suboxone | 3A4 |
| telithromycin | 3A4 |
| boceprevir | 3A4 |
| cobicistat | 3A4 |
| darunavir | 3A4 |
| delavirdin | 3A4 |
| idelalisib-metabolite GS-563117 | 3A4 |
| nelfinavir | 3A4 |
| posaconazol | 3A4 |
| telaprevir | 3A4 |
| troleandomycin | 3A4 |
| voriconazol | 3A4 |
| erythromycin | 3A4 |
